# The FcγRIII Engagement Augments PMA-Stimulated Neutrophil Extracellular Traps (NETs) Formation by Granulocytes Partially via Cross-Talk between Syk-ERK-NF-κB and PKC-ROS Signaling Pathways

**DOI:** 10.3390/biomedicines9091127

**Published:** 2021-09-01

**Authors:** Cheng-Hsun Lu, Ko-Jen Li, Cheng-Han Wu, Chieh-Yu Shen, Yu-Min Kuo, Song-Chou Hsieh, Chia-Li Yu

**Affiliations:** 1 Department of Internal Medicine, National Taiwan University Hospital, Taipei 10002, Taiwan; b89401085@ntu.edu.tw (C.-H.L.); dtmed170@gmail.com (K.-J.L.); chenghanwu@ntu.edu.tw (C.-H.W.); tsichhl@gmail.com (C.-Y.S.); 543goole@gmail.com (Y.-M.K.); hsiehsc@ntu.edu.tw (S.-C.H.); 2 Graduate Institute of Clinical Medicine, National Taiwan University College of Medicine, Taipei 10002, Taiwan

**Keywords:** polymorphonuclear neutrophil, neutrophil extracellular traps, differentiated HL-60 cells, IgG subclass, FcγRIII engagement, reactive oxygen species, Syk-ERK signaling pathway, PAD4

## Abstract

Polymorphonuclear neutrophils (PMNs) are the most abundant white blood cell in the circulation capable of neutrophil extracellular traps (NETs) formation after stimulation. Both NADPH oxidase-dependent and -independent pathways are involved in NET formation. The IgG is the most abundant immunoglobulin in human serum. However, the impact of the circulating IgG on NET formation is totally unexplored. In this study, the all-trans retinoic acid (ATRA)-induced mature granulocytes (dHL-60) were pre-treated with monomeric human IgG, papain-digested Fab fragment, crystallizable IgG Fc portion, rituximab (a human IgG1), or IgG2. The NET formation of the dHL-60 in the presence/absence of phorbol 12-myristate 13-acetate (PMA) stimulation was then measured by the fluorescent area after SYTOX green nucleic acid stain. The intracellular reactive oxygen species (ROS) generation was measured by flow cytometry. Total and phosphorylated Syk, SHP-1, and ERK were detected by immunoblot. We found that human monomeric IgG and its subclasses IgG1 and IgG2 per se induced negligible NET formation of dHL-60, but the FcγRIII engagement by these IgG subclasses and Fc portion augment PMA-stimulated dHL-60 NET formation in a dose-dependent manner. Furthermore, we found that increased Syk and ERK phosphorylation, intracellular ROS generation, and pro-inflammatory cytokines, IL-8 and TNF-α, production could be induced after FcγRIII engagement. Blocking FcγRIII engagement by a specific antibody diminished the augmented NET formation. In conclusion, we discovered that cross-talk between FcγRIII engagement-induced Syk-ERK and PMA-induced PKC signaling pathways augment NET formation of dHL-60 via increased ROS generation and pro-inflammatory cytokines, IL-8 and TNF-α, production.

## 1. Introduction

Human polymorphonuclear neutrophils (PMNs) are the most abundant leukocytes in human blood [1]. PMNs play a pivotal role in innate immunity with a high potency and efficacy to sense and then eradicate microbial infections [2]. Among these anti-microbial activities, the most effective mechanism is the extrusion of the intracellular material in the form of neutrophils extracellular traps (NETs) into the surrounding milieu [3,4,5]. NETs can effectively trap the invading pathogens for preventing spreading [6]. Besides, many granule anti-microbial molecules such as elastase, proteinase, myeloperoxidase, and LL-37 attached on the extruded DNA threads can rapidly degrade the virulence factors and kill bacteria [6,7,8].

**Figure 1:**
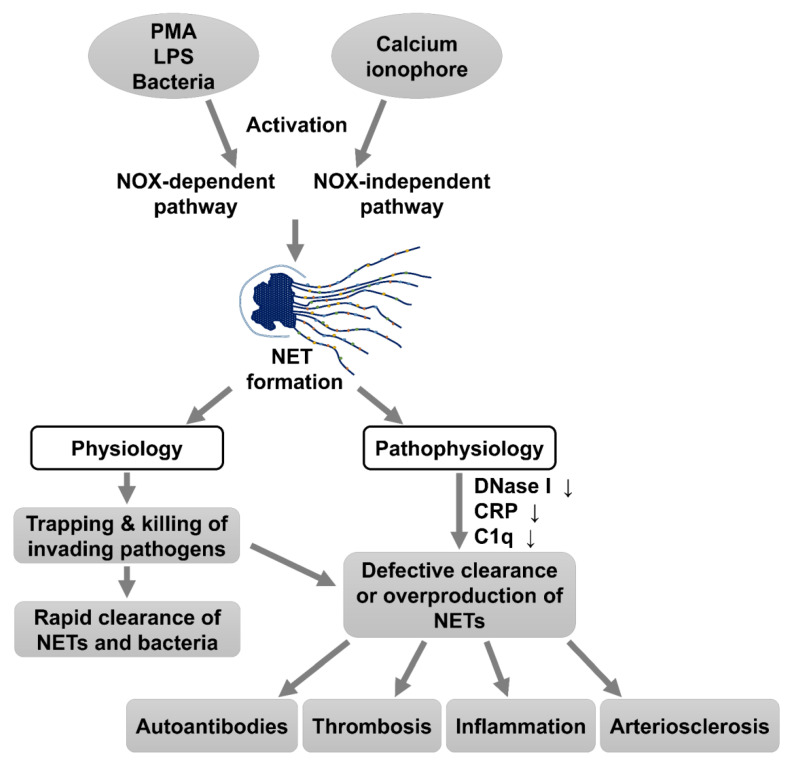
The induction and physiological/pathophysiological roles of NET formation in human diseases. Both NADPH oxidase (NOX)-dependent and -independent pathways are induced by different chemicals, bacteria, or molecules for NET formation. The physiological functions of NET formation aim to rapidly trap and kill the invading pathogens followed by clearance of NET via DNase I, C-reactive protein (CRP), and C1q. However, in some pathophysiological conditions such as the deficiency of these serum molecules or excessive NET formation, the released DNA, histone, and granule proteins become the neoantigens to stimulate autoantibody production, inflammation, and platelet entrapment. Subsequently, thrombosis, arteriosclerosis, and autoimmunity occur.

NETs formation can be triggered by a diverse stimuli including phorbol 12-myristate 13-acetate (PMA), protein kinase C (PKC), lipopolysaccharide (LPS), bacteria, uric acid crystals, and calcium ionophores [6,9,10,11]. Regardless of the stimuli, NETs can be initiated by intracellular reactive oxygen species (ROS) and high concentration of [Ca^2+^] [7,12,13]. Arbitrarily, both NADPH oxidase (NOX)-dependent and NOX-independent pathways are involved in the intracellular ROS generation [3,9,14]. In addition, peptidylarginine deiminase 4 (PAD4) is required in both pathways for mediating arginine citrullination of histones to initiate chromatin decondensation and nuclear envelope rupture [3,14,15,16]. Immediately after NET formation, DNase I, C-reactive protein (CRP), and complement C1q can facilitate the degradation and clearance of NET products [3,17,18,19]. The imbalance of NETs formation and degradation may exert detrimental effects to the immune responses [19] because NETs contain many sources of autoantigens. Accordingly, the dysregulation in NET formation and/or defective clearance participates in the development and flare-up of various autoimmune diseases [8,20,21,22,23]. A scheme outlining the generation and the physiological/pathophysiological roles of NET formation is demonstrated in Figure 1.

Anti-neutrophil cytoplasmic antibodies (ANCAs) are considered the important autoantibodies to stimulate PMN activation and then NETosis (a kind of PMN apoptosis after NET formation), and potentially exert positive feed-back to ANCA formation, which correlate with vasculitis activity [24]. When NET molecules are massively released and cannot be cleared, a range of pathological conditions including vascular thrombosis, atherosclerosis, autoimmune diseases, and sepsis occur [19,21,25,26]. Recently, the pandemic SARS-CoV-2 infection was found able to activate NETosis of human PMNs. The abundant NET formation with increased production of intracellular reactive oxygen species (ROS) led to thrombosis formation and lung epithelial cell injury [27,28,29]. Colchicine is found to be an inhibitor of NETosis [30,31], and a recent randomized controlled trial demonstrated that colchicine reduced the length of both supplemental oxygen therapy and hospitalization [32]. Thus, further investigation to explore the crucial factors in regulating NETosis is important for managing various types of autoimmune and inflammatory diseases.

Among the different immunoglobulin (Ig) isotypes, IgA has been reported to enhance NETs formation via Fcα receptor I on PMNs [33,34]. However, the concentration of immunoglobulin G (IgG) is the highest among the Ig isotypes in the human serum. Intravenous immune globulin (IVIg) therapy has been shown to be effective in certain autoimmune and inflammatory diseases [35]. It is believed that a number of immune effects are mediated through Fcγ receptor (FcγR) engagement after IVIg therapy [36]. Nevertheless, the effects of IVIg may differ depending on different diseases [37,38]. Among the four human Ig subclasses IgG1-IgG4, IgG1 and IgG2 are the most abundant. Ying et al. [39] found that monomeric IgG1 Fc fragment displays unique FcγR interactions that are exploitable to treat inflammation-mediated diseases. However, the impact of circulating IgGs on NET formation has not been reported in the literature. Jönsson et al. [40] demonstrated that an IgG-induced neutrophil activation pathway contributes to drug-induced anaphylaxis via enhancing NETosis. Recently, Fetz et al. [41] found that IgG adsorbed by electrospun polydioxanone biomaterials can engage FcγRIIIB and is responsible for biomaterial-induced NETosis of human neutrophils.

Despite the above findings, to our knowledge, there was no study on the interactions between human IgG monomer and NET formation. We hypothesize that the circulating IgG can modify neutrophil functions by altered NET formation via the engagement of FcγR. In considering the short lifespan of human neutrophils in the circulation and in vitro [42], the mature human granulocytes derived from all-trans retinoic acid-induced (ATRA) differentiated HL-60 cells (dHL-60) were adopted in this study [43,44]. The effects and the possible molecular mechanisms of human IgG subclasses on PMA-stimulated dHL-60 NET formation were explored in the present study.

## 2. Materials and Methods

### 2.1. Cell Culture and Induction of HL-60 Cell Differentiation

HL-60 cells (a human promyelocytic leukemia cell line, CCL240, American Type Culture Collection, Rockville, MD, USA) suspended in RPMI-1640 medium (Gibco, Thermo Fisher Scientific, Waltham, MA, USA) supplemented with 10% (*v/v*) heat-inactivated fetal bovine serum (hereafter referred to as complete culture medium) were cultured at 37 °C in 5% CO_2_–95% air. Induction of HL-60 differentiation was achieved by seeding 10^6^ cells/mL in 24-well plates in the presence of a final concentration of 10 μM of all-trans retinoic acid (ATRA, Sigma-Aldrich, St. Louis, MO, USA) and 25 ng/mL of G-CSF (PeproTech, Rocky Hill, NJ, USA) for 5 days.

After induction, the differentiated HL-60 cells (dHL-60) were confirmed to become mature granulocytes by the surface expression of CD11b and CD16. In brief, cells were doubly stained with PerCP-Cy5.5-conjugated CD11b antibody (BD Biosciences, Oxford, UK) and PE-conjugated CD16 antibody (BD Biosciences, Oxford, UK), followed by FACSLyric flow cytometer analysis (BD Life Sciences, San Jose, CA, USA).

### 2.2. Incubation of Human Monomeric IgG1, IgG2, and the Fab and Fc Fragments of Human IgG with dHL-60

Native human monomeric IgG1 (ab90283), IgG2 (ab90284), papain-digested IgG Fab fragment (ab90352), and IgG Fc fragment (ab205805) were purchased from Abcam (Cambridge, MA, USA). The IVIg was prepared from pooled human plasma (TBSF, Taipei, Taiwan) as surrogate human IgG molecule and rituximab (RTX) as human surrogate IgG1 was obtained from Roche Pharmaceutical Company (Basel, Switzerland). These molecules were diluted in complete culture medium. The dHL-60 cells were incubated with various doses of the monomeric IgG molecules at 37 °C for variable times according to the experimental design.

### 2.3. Measurement NETs Formation

The dHL-60 cells at a concentration of 2 × 10^6^ cells/mL were seeded into channel slides with a channel volume of 30 µL (µ-Slide VI 0.4, ibidi, Gräfelfing, Germany) that allows fluorescence imaging of live cells. PMA obtained from Sigma-Aldrich (St. Louis, MO, USA) at a final concentration of 100 nM was added for stimulating NET formation at 37 °C in 5% CO_2_–95% air for 4 h. SYTOX green nucleic acid stain solution (Invitrogen, Carlsbad, CA, USA) was added at a final concentration of 250 nM for extracellular DNA detection. The fluorescence of channel slide was observed by fluorescence microscope. The decondensed DNA expulsed into the extracellular space can be read without fixation. For quantitation, four pictures in quarters of each channel are calculated for fluorescent area (NET formation) using the macro script of ImageJ version 1.52p (National Institutes of Health, Bethesda, MD, USA) as described by Rebernick et al. [45]. The ratio of fluorescent area is calculated by dividing the fluorescence of the PMA stimulation alone. For further confirming the NET formation indeed by dHL-60, the channel slides were stained with: (1) DAPI solution (1:1000, Invitrogen, Carlsbad, CA, USA), and (2) rabbit anti-histone H3 (citrulline R2 + R8 + R17) antibody (1:250, Abcam, Cambridge, MA, USA) overnight and then the cells were stained with goat anti-rabbit Alexa Fluor 594-conjugated secondary antibody (1:500, Invitrogen, Carlsbad, CA, USA) for 1 h. After washes, the NETs formation was further confirmed by positive staining for both citrullinated histone and DNA in addition to extracellular green fluorescence of DNA.

Besides, monoclonal anti-human CD16 antibody (Biolegend, San Diego, CA, USA) was added at a concentration of 5 μg/mL to dHL-60 cell suspension for 10 min at 37 °C in 5% CO_2_–95% air for blocking the FcγRIII before incubation with different IgG molecules.

### 2.4. Determination of ROS Generation by PMA-Stimulated dHL-60 Cells

#### 2.4.1. Quantification of NADPH/NADP^+^ Ratio

The ratio of NADPH/ NADP^+^ was measured for assessing the intracellular redox potential or capacity. To determine the intracellular NADPH/NADP^+^ ratio, the quantification kit (Sigma-Aldrich, St. Louis, MO, USA) was applied for the detection. Briefly, the dHL-60 at a concentration of 2 × 10^6^ cells/mL were suspended in glucose-free RPMI-1640 medium (Gibco, Thermo Fisher Scientific, Waltham, MA, USA) supplemented with 10% (*v/v*) heat-inactivated fetal bovine serum, and then incubation with IgG molecules (100 μg/mL), RTX (100 μg/mL), or LPS obtained from *Pseudomonas aeruginosa* (0.1 μg/mL, Sigma-Aldrich, St. Louis, MO, USA) for 0.5 h at 37 °C in 5% CO_2_–95% air. After centrifugation at 2000 RPM at 4 °C for 5 min, the cell pellets were lysed by adding 400 μL extraction buffer for 10 min on ice, followed by centrifuging at 10,000× *g* for 10 min. The supernatants were filtered through a 10 kDa spin column (Merck KGaA, Darmstadt, Germany). The concentration of NADPH and total NADP^+^ were measured according to the manufacturer’s instructions.

#### 2.4.2. Measurement of Nitric Oxide

The dHL-60 cells at a concentration of 2 × 10^6^ cells/mL were incubated with Fab or Fc fragment of human monomeric IgG (100 μg/mL) overnight at 37 °C in 5% CO_2_–95% air. After incubation, the supernatant was collected by centrifuging at 16,000× *g* for 20 min at 4 °C. The Griess reagent nitrite measurement kit (Cell Signaling Technology, Danvers, MA, USA) was used to indirectly detect the concentration of nitric oxide through measuring one of its stable oxidation products, nitrite, according to the manufacturer’s instructions.

#### 2.4.3. Assessment of the General ROS in Cells

The dHL-60 were incubated with IgG (200 μg/mL), IgG1 (200 μg/mL, RTX), or LPS (1 μg/mL) for 1 h in complete culture medium at 37 °C. Monoclonal anti-human CD16 antibody (5 μg/mL) was added 10 min before incubation with IgG molecules to block the FcγRIII. The intracellular ROS was measured by using the fluorescent probe 5-(and-6)-chloromethyl-2′,7′-dichlorodihydrofluorescein diacetate, acetyl ester (CM-H2DCFDA, Invitrogen, Carlsbad, CA, USA). After loading the ROS-sensitive probe CM-H2DCFDA, the cells were incubated at 37 °C with or without PMA (20 nM) for 20 min followed by several washes by PBS. Fluorescence was measured by a FACSLyric flow cytometer system with excitation of 488 nm and emission of 527 nm. To measure the shift in fluorescence intensity, the geometric mean (GeoMean) fluorescence was obtained for comparison.

### 2.5. Western Blot Analysis

dHL-60 cells were incubated with IgG (200 μg/mL), RTX (200 μg/mL), Fc fragments (70 μg/mL), LPS (1 μg/mL), or PMA (100 nM) for 5, 10, and 20 min in complete culture medium at 37 °C. The cells were then lysed in RIPA buffer supplemented with protease inhibitor cocktail (Roche Applied Science, Penzberg, Germany). The lysates were then electrophoresed in 10% SDS-PAGE and transferred onto polyvinylidene difluoride membrane (Millipore, Billerica, MA, USA). The membranes were probed with rabbit anti-p44/42 MAPK (Erk1/2) antibody (Cell Signaling Technology, Danvers, MA, USA), rabbit anti-phospho-p44/42 MAPK (Erk1/2) antibody (Thr202/Tyr204) (Cell Signaling Technology, Danvers, MA, USA), rabbit anti-phospho-Syk (Tyr525/526) antibody (Cell Signaling Technology, Danvers, MA, USA), mouse anti-Syk antibody (Cell Signaling Technology, Danvers, MA, USA), rabbit anti-phospho-SHP-1 (Tyr564) antibody (Cell Signaling Technology, Danvers, MA, USA), and rabbit anti-SHP-1 antibody (Cell Signaling Technology, Danvers, MA, USA), while mouse anti-glyceraldehyde-3-phosphate dehydrogenase (GAPDH) (Sigma-Aldrich, St. Louis, MO, USA) was used as internal control. After washes, the conjugates were then stained with horseradish peroxidase-conjugated secondary antibodies. Image densitometry was analyzed by ImageJ version 1.52p (National Institutes of Health, Bethesda, MD, USA).

### 2.6. Quantitation of Proinflammatory Cytokines

Both HL-60 and dHL-60 cells at a concentration of 2 × 10^6^ cells/mL were incubated with IgG (200 μg/mL), RTX (200 μg/mL), or LPS (0.1 μg/mL) in complete culture medium at 37 °C overnight. Monoclonal anti-human CD16 antibody (1 μg/mL) was added 10 min before incubation with IgG molecules to block the FcγRIII. The concentration of IL-8 and TNF-α in the cultured supernatants was assessed by the respective ELISA kit (R&D Systems, Minneapolis, MN, USA) according to the manufacturer’s instructions.

### 2.7. Statistical Analyses

Data were statistically analyzed by Mann–Whitney and Wilcoxon rank sum tests using GraphPad Prism 8.0.2 (GraphPad Software Inc, San Diego, CA, USA). Spearman’s rank correlation coefficient was calculated via software R version 4.0.5 (R Foundation for Statistical Computing, Vienna, Austria). All experiments were repeated at least three times.

## 3. Results

### 3.1. PMA-Stimulated NET Formation by dHL-60

**Figure 2:**
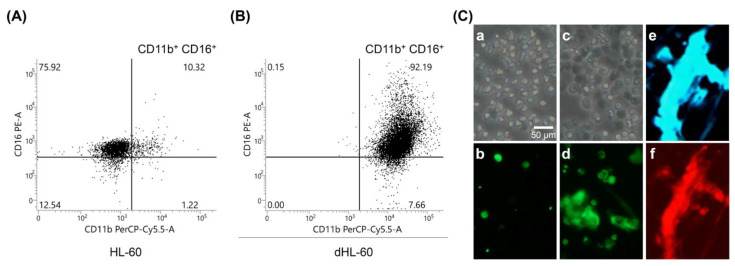
The induction of human promyelocytic leukemia cells (HL-60) into mature granulocytes (dHL-60) by all-trans retinoic acid (ATRA), and the PMA-stimulated NET formation by dHL-60 cells. (**A**) The expression of CD11b^+^CD16^+^ surface markers on undifferentiated HL-60 cells detected by two-color flow cytometry. Only 10.32% cells are double positive. (**B**) The expression of CD11b^+^CD16^+^ surface markers on ATRA-induced dHL-60 cells. After ATRA induction, 92.19% of dHL-60 express both CD11b and CD16 granulocyte differentiation markers on the cell surface. (**C**) A representative case showing NET formation in PMA-activated dHL-60. (**a**) Light microscopic observation of dHL-60. (**b**) SYTOX green nucleic acid stain of non-stimulated dHL-60 cells observed by fluorescent microscope. (**c**) Light microscopic observation of 4 h PMA-stimulated dHL-60. (**d**) SYTOX green nucleic acid stain of 4 h PMA-stimulated dHL-60 observed by fluorescent microscope. (**e**) NET formation of PMA-stimulated dHL-60 identified by DAPI stain. (**f**) NET formation of PMA-stimulated dHL-60 identified by Alexa Fluor 594-labeled anti-citrullinated histone H3 antibody stain. Scale bar: 50 μm.

Mature granulocytes are identified by the double expression of CD11b and CD16 on the cell surface. Before differentiation induction, there was only 10.23% of double biomarker positivity on undifferentiated HL-60 cells (Figure 2A). In contrast, the percentage of CD11b^+^CD16^+^ dHL-60 was found high up to >90% after induction, indicating that a high proportion of HL-60 cells were differentiated into mature granulocytes as shown in Figure 2B. The NET formation of dHL-60 induced by PMA stimulation is demonstrated in Figure 2C. Compared to the unstimulated cells (Figure 2C(a,b)), the formation of NETs in PMA-stimulated dHL-60 cells (Figure 2C(c,d)) was shown by increased SYTOX green nucleic acid stain. The increased generation of NETs was further confirmed by a double stain of extracellular DNA by DAPI (Figure 2C(e)) and citrullinated histone H3 (Figure 2C(f)). Besides, the amount of NET formation can be quantified by measuring the fluorescent area of the extracellular DNA (Figure 2C(d)) by ImageJ.

### 3.2. The Effects of Preincubation of dHL-60 with Different Human Monomeric IgG Subclasses (IgG1 and IgG2) and IgG Fragments (Fab and Fc) on PMA-Stimulated NET Formation

**Figure 3:**
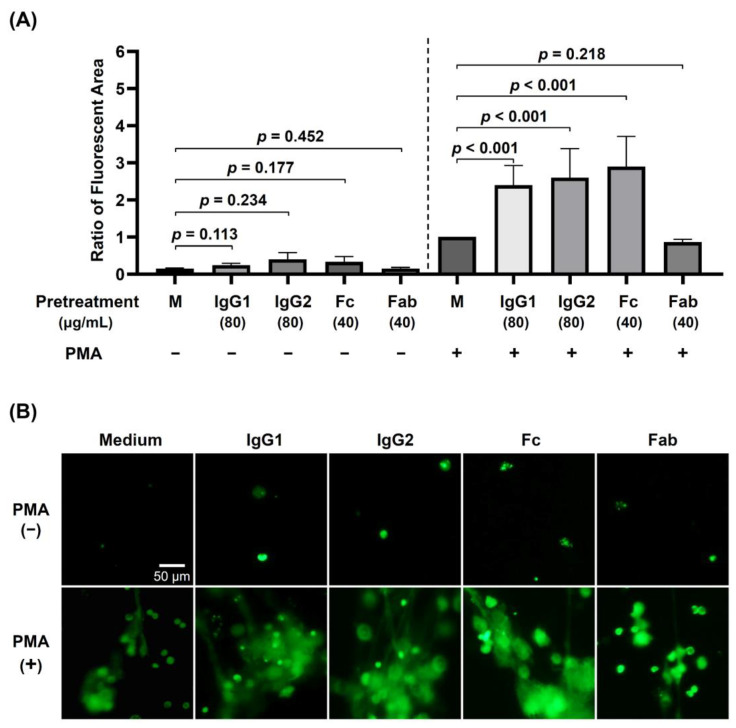
The effects of pretreatment of human monomeric IgG subclasses (IgG1 and IgG2) and IgG fragments (Fab and Fc) on PMA-activated dHL-60 NET formation. (**A**) The NET formation represented by green fluorescence area is detected by SYTOX green nucleic acid stain. The left panel shows without PMA stimulation whereas the right panel shows with PMA stimulation. For comparison, the ratio of fluorescent area is calculated by the individual fluorescence area divided by the PMA-stimulation with medium preincubation (M). (**B**) A representative case showing the NET formation of dHL-60 pretreated with different molecules and then without (upper panel) or with (lower panel) PMA stimulation. Compared to medium control, the fluorescence areas are augmented in IgG1, IgG2, and IgG Fc-pretreated, but not in Fab-pretreated PMA-activated dHL-60 granulocytes. M: medium. Scale bar: 50 μm.

Incubation of human monomeric IgG1, IgG2, IgG Fab, or IgG Fc fragment with dHL-60 cannot significantly induce NET formation by non-PNA-stimulated-dHL-60 (Figure 3A, left panel). However, the pre-incubation of these IgG molecules, except Fab fragment, can significantly augment the PMA-induced fluorescent area (denotes NETs formation) by dHL-60 (Figure 3A, right panel). These results may indicate that the engagement of FcγR can tremendously enhance PMA-stimulated NET formation of dHL-60. A representative case is presented by measuring the fluorescence areas in the absence (upper panel) and presence (lower panel) of PMA-stimulation after FcγR engagement in Figure 3B.

### 3.3. Dose-Dependent Augmenting Effect of Human IgG Fc Receptor Type III (FcγRIII) Engagement on PMA-Stimulated dHL-60 NET Formation

**Figure 4:**
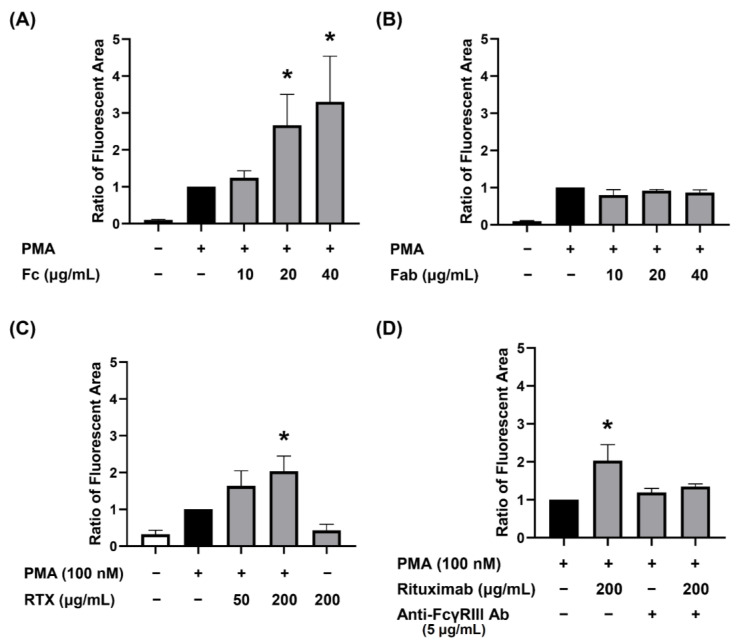
Dose-dependent augmenting effect of Fc, but not Fab, fragment derived from human IgG and IgG1 (rituximab, a human chimeric monoclonal IgG1 antibody) on PMA-stimulated dHL-60 NET formation via FcγRIII engagement. The NET area was detected by SYTOX green nucleic acid stain and was denoted by the ratio compared to PMA stimulation only. (**A**) Dose-dependent augmentation (10–40 μg/mL) of human IgG Fc fragment engagement on PMA-stimulated dHL-60 NET formation. (**B**) Negligible augmenting effect of human IgG Fab fragment from 10–40 μg/mL on PMA-stimulated dHL-60 NET formation. (**C**) Dose-dependent augmentation of rituximab (RTX, an IgG1 monoclonal antibody, 50–200 μg/mL) on PMA-stimulated dHL-60 NET formation. (**D**) The anti-FcγRIII antibody (5 μg/mL) pretreatment can suppress the RTX-augmented PMA-stimulated dHL-60 NET formation. * *p* < 0.05 compared to PMA only.

In the stimulation of PMA, human IgG Fc fragment (from 10–40 μg/mL) dose-dependently augmented NET formation compared to PMA alone as shown in Figure 4A. In contrast, IgG Fab fragment (from 10–40 μg/mL) failed to enhance the effect (Figure 4B). Hunan IgG1 (represented by RTX) also showed the dose-dependent augmentation in the presence of PMA stimulation (Figure 4C). We then used a specific anti-FcγRIII antibody to block the IgG antibody engagement in advance. The augmentation by IgG1 molecule (RTX) was abolished in PMA-stimulated dHL-60 NET formation (Figure 4D). These results may further confirm that the engagement of IgG Fc receptors really augments NET formation by PMA stimulation.

### 3.4. Increased Intracellular ROS Production and ROS Released from dHL-60 via FcγRIII Engagement

#### 3.4.1. Intracellular NADPH/NADP^+^ Ratio in dHL-60 and Nitrite in the Culture Supernatants without PMA Stimulation

**Figure 5:**
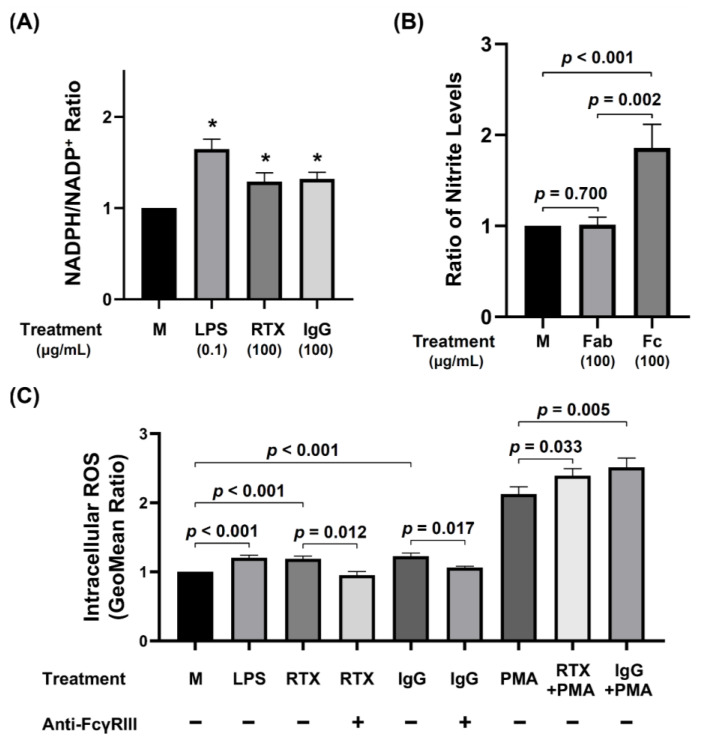
Increased dHL-60 ROS production by monomeric IgG and rituximab (RTX, IgG1 subclass) Fc region engagement per se. (**A**) Compared to medium control (M), RTX and normal human IgG per se significantly enhanced NADPH/NADP+ ratio. (**B**) IgG Fc fragment (100 μg/mL), but not Fab fragment (100 μg/mL), engagement significantly enhanced nitrite production from dHL-60. (**C**) Anti-FcγRIII antibody (5 μg/mL) pretreatment significantly inhibits IgG (200 μg/mL) and RTX (200 μg/mL)-enhanced intracellular ROS generation. In addition, the engagement of IgG and RTX can augment PMA-stimulated intracellular ROS generation by dHL-60. * *p* < 0.05 compared to the medium control (M).

The intracellular NADPH/NADP^+^ ratio in dHL-60 was enhanced by human IgG and IgG1 as well as LPS per se (Figure 5A). The extracellular nitrite concentration in the culture supernatant was also higher in dHL-60 after overnight incubation with IgG Fc fragments (Figure 5B). In contrast, incubation with Fab fragments did not change nitrite concentration in the supernatant. These results may suggest that the engagement of FcγRIII can change intracellular redox reaction toward oxidative state.

#### 3.4.2. Increased Intracellular ROS Concentration in dHL-60 Cells by IgG Molecules per se via Fc Region Engagement

Incubation of dHL-60 cells with monomeric human IgG or RTX along can induce higher intracellular ROS production than medium. The increment can be abrogated by previous incubation with anti-FcγRIII antibody (Figure 5C). These results may further confirm that increased intracellular ROS generation of dHL-60 cells by human IgG or IgG1 molecule per se is through FcγRIII engagement. 

### 3.5. Activation of Syk-ERK Signaling Pathway by IgG Fc Receptor Engagement

**Figure 6:**
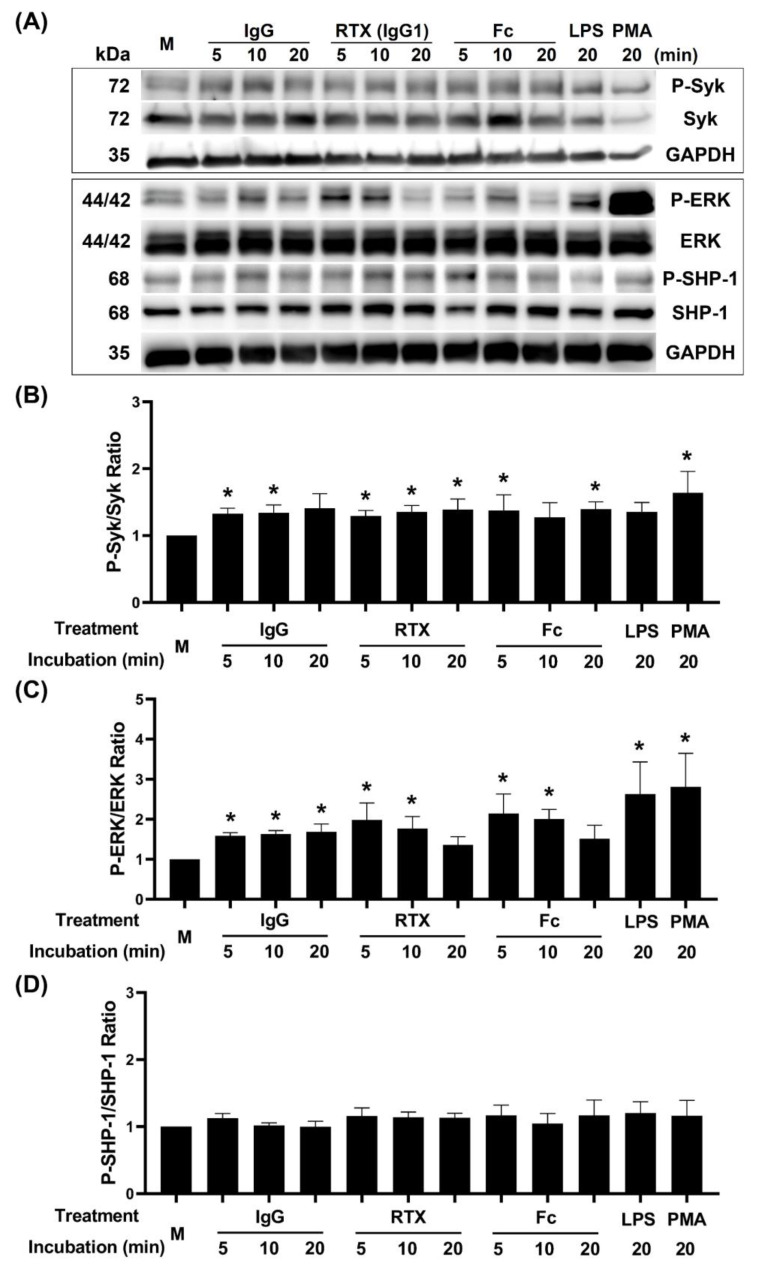
Activation of Syk-ERK, but not SHP-1, signaling pathway by IgG Fc fragment engagement per se. (**A**) A representative case of Western blot detection of P-Syk, P-ERK, and P-SHP-1 expression in IgG (200 μg/mL), IgG1 (RTX 200 μg/mL), and IgG Fc fragment (70 μg/mL)-engaged dHL-60 compared to LPS (1 μg/mL) and PMA (100 nM) stimulation. (**B**) Statistical analysis of P-Syk/total Syk ratio in different groups by five experiments. (**C**) Statistical analysis of P-ERK/total ERK ratio in different groups by five experiments. (**D**) Statistical analysis of P-SHP-1/total SHP-1 ratio in different groups by five experiments. * *p* < 0.05 compare to medium control (M).

For further investigating the molecular mechanisms of FcγRIII engagement enhancing PMA-stimulated dHL-60 NET formation, the intracellular signal molecules including phosphorylated (P)-Syk, P-ERK, and P-SHP-1 were detected by Western blot. We repeated the assay at least five times and a representative case is demonstrated in Figure 6A. For quantitation, the ratio of P-Syk/total Syk (Figure 6B), P-ERK/ERK (Figure 6C) and P-SHP-1/SHP-1 (Figure 6D) were calculated in different incubation time periods. These data indicate that the FcγRIII engagement by human IgG, IgG1, and IgG Fc fragment enhance the phosphorylation of Syk-ERK, but not SHP, to transduce the Syk-ERK-NF-κB interior signaling pathway.

### 3.6. The Release of Proinflammatory Cytokines by dHL-60 Cells

**Figure 7:**
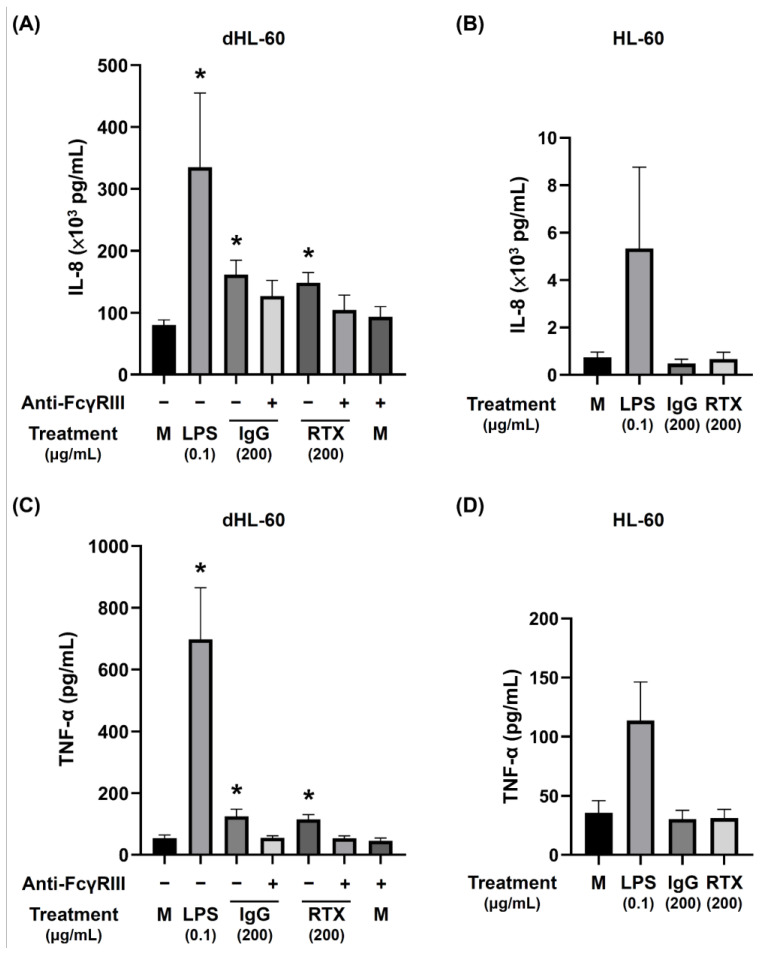
Increase in IL-8 and TNF-α production by dHL-60, but not by undifferentiated HL-60, after IgG and rituximab (RTX, IgG1 subclass) engagement. (**A**) Increased IL-8 production by dHL-60 cells after engagement by IgG and RTX that can be abolished by anti-FcγRIII antibody (1 μg/mL). (**B**) No increased IL-8 production by undifferentiated HL-60 cells. (**C**) Increased TNF-α production by dHL-60 cells after engagement by IgG and RTX that can be abolished by anti-FcγRIII antibody (1 μg/mL). (**D**) TNF-α production by undifferentiated HL-60 cells. * *p* < 0.05 compare to medium control (M).

The release of proinflammatory cytokines, IL-8 from dHL-60, into the culture supernatants were significantly increased after incubation with IgG and RTX overnight per se. The anti-FcγRIII antibody (1 μg/mL) pretreatment can suppress the cytokines secretion stimulated by IgG and RTX (Figure 7A). In contrast, the production of IL-8 by non-differentiated HL-60 was not enhanced by IgG or RTX as shown in Figure 7B. The situation of TNF-α production by dHL-60 (Figure 7C) vs. HL-60 (Figure 7D) was the same as in IL-8. These results indicate that the proinflammatory cytokine production was enhanced by IgG and RTX requires FcγRIII engagement. The increased IL-8 and TNF-α by FcγRIII engagement seems not enough to induce dHL-60 NET formation. However, in synergy with PMA-induced proinflammatory cytokines production, the dHL-60 NET formation can be augmented as shown in the right panel of Figure 3A.

## 4. Discussion

In this study, we originally found that human purified monomeric IgG subclasses IgG1 and IgG2, and IgG Fc fragment can augment PMA-stimulated NET formation of dHL-60 cells via the engagement of FcγRIII. We used the ATRA-differentiated HL-60 cells (mature granulocytes) cultured in human immunoglobulin-free medium instead of isolated human peripheral blood PMNs. Therefore, we can completely control the experimental milieu to prevent previous engagement of dHL-60 by human IgGs and interference for NET formation. It is interesting that the engagement of FcγRIII by human IgG1, IgG2, and Fc portion of IgG can enhance intracellular ROS (Figure 5) and proinflammatory cytokines, IL-8 and TNF-α (Figure 7), but is unable to induce NET formation of dHL-60. Unexpectedly, the engagement with IgG subclasses or IgG Fc fragments can augment PMA-activated NET formation of dHL-60 cells in a dose-dependent manner. It is worthy to mention that the concentrations of IgG subclasses we used to enhance PMA-stimulated dHL-60 NET formation are much lower than those in human serum. This finding may suggest that the FcγR engagement already happens to influence NET formation in vivo. In clinical practice, Yalavarthi et al. [46] found that IgG purified from anti-phospholipid syndrome patients can stimulate NET formation by normal neutrophils with the requirement of ROS. Our findings of increased ROS generation by FcγRIII engagement of dHL-60 is rather consistent with this observation and may provide some novel insights with the mechanism. For further exploring the involvement of IgG Fc receptors in PMA-stimulated NET formation, the signaling pathways induced by IgG Fc receptor engagement and the molecules involved in NET formation were investigated in the present study.

**Figure 8:**
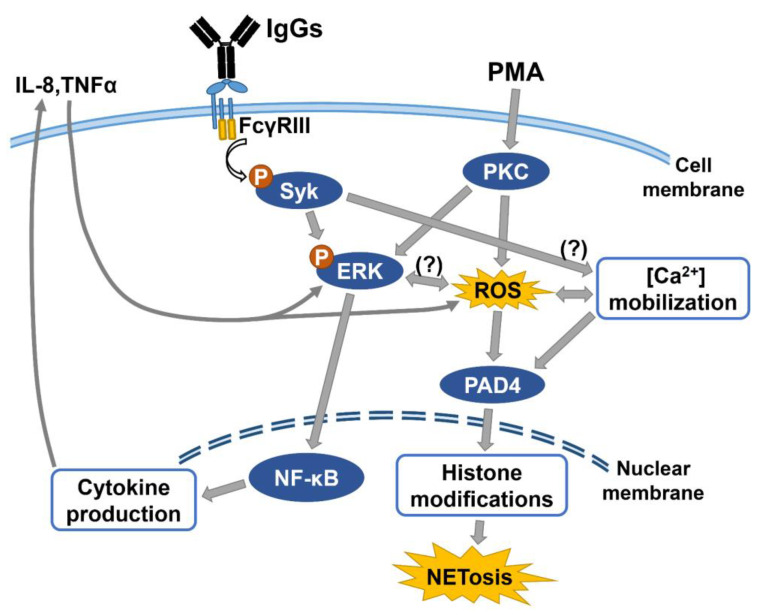
A putative scheme demonstrating the cross-talk between FcγRIII engagement-induced Syk-ERK-NF-κB and PMA-induced PKC-ROS signaling pathways in augmenting dHL-60 NET formation. Human monomeric IgG binding to FcγRIII activates Syk-ERK- NF-κB signaling pathway, which enhances intracellular ROS generation and IL-8 and TNF-α cytokines gene expression. These cytokines can positively feedback the ERK-NF-κB signaling and ROS generation. The ROS and high concentration of intracellular [Ca^2+^] can stimulate PAD4 enzyme activity for histone citrullination. On the other side, the PMA-activates PKC phosphorylation can potently induce ROS generation and PAD4 activation. It is quite possible that the cross-talk between FcγRIII-Syk-ERK and PMA-PKC signaling pathways can synergistically augment NET formation. PAD4: peptidylarginine deiminase 4.

As demonstrated in Figure 6 and summarized in Figure 8, we propose that FcγR engaged by human IgG Fc fragment can activate Syk-ERK-NF-κB signaling to enhance ROS generation, which is crucial for NET formation [7,12,13]. However, the enhanced intracellular ROS are not as high as that induced by PMA. One possible explanation may be in the slightly increased P-SHP-1 after the FcγR engagement (Figure 6D) via FcγRII [47]. It is believed that P-SHP-1 can down-regulate the Syk-ERK signaling pathway to suppress the cell activation [47,48]. Although the Fc region of IgG alone cannot trigger NET formation of dHL-60, we originally found that the FcγR engagement can augment NET formation of dHL-60 in the presence of PMA via increased ROS and proinflammatory cytokines, IL-8 and TNF-α, production. On the other hand, PMA can activate the PKC signaling pathway to enhance ROS generation [49,50]. Obviously, the partial cross-talk between FcγRIII-induced Syk-ERK signaling pathway and PMA-induced PKC signaling pathway is directly via ROS generation, and indirectly via proinflammatory cytokines, IL-8 and TNF-α, production. Durandy et al. [51] demonstrated that the proinflammatory property of IgG requires binding of the IgG Fc fragment to FcγR on innate immune effector cells. Some authors have demonstrated that the interaction between IgG Fc portion and FcγRIII could induce proinflammatory cytokines and phagocytosis of target cells [52,53]. Although we did not perform an experiment to test the effects of IL-8 and TNF-α on granulocytes, it is already proved that proinflammatory cytokines, IL-8 and TNF-α, can influence NET formation via a positive feedback to ERK pathway and ROS production [54,55,56,57].

The HL-60 cell line originated from a patient with acute promyelocytic leukemia. Our finding of the production of IL-8 and TNF-α from myeloid cells after FcγR engagement can be applied in the pathophysiology of a notable clinical condition. The differentiation syndrome is a serious complication in patients with acute promyelocytic leukemia after receiving the treatment regimens of ATRA [58,59]. This particular syndrome is associated with the presentation of organ infiltration by granulocytes and those mature white blood cells activated by the inflammatory cytokines. It is also conceivable that the proinflammatory cytokines can induce a systemic inflammatory response syndrome, the main phenomenon in this particular syndrome [60]. Although the pathogenesis of differentiation syndrome is still not completely understood, the up-regulated proinflammatory cytokines, IL-8 and TNF-α, production by dHL-60 upon engagement of FcγRIII seems not only consistent with the development of differentiation syndrome but enhanced PMA-stimulated NET formation via ROS generation.

We used several types of IgG molecules containing the Fc region, including monomeric IgG1 (RTX), IgG2, and Fc fragments derived from human IgG in this study. Since IgG1 is the most abundant IgG in human serum, it is worthy to investigate its role in NET formation. As a chimeric mouse-human IgG1 monoclonal antibody, RTX had the advantages of stable structure and being easily available. The IgG we used in the present study was purified from the pooled plasma of healthy blood donors in Taiwan, whereas the Fc fragment was obtained from the normal human plasma of Caucasians. The Fc glycosylation may differ relying on the origin, especially after papain digestion. Therefore, the FcγRIII engagement of IgG and Fc fragments may produce unequal degrees of immunological effects. Nevertheless, we found the effects of different Fc region-containing IgG molecules to be rather consistent with the results obtained.

Human neutrophils can express both FcγRIIIA and FcγRIIIB on the cell surface [36,41,61]. FcγRIIIB is well known as the most abundant receptor on the surface of mature neutrophils [62,63]. Human neutrophils also express low levels of FcγRIIIA, which plays a role in PMN activation [61,63]. The phosphorylation of Syk can be induced by both FcγRIIIA and FcγRIIIB engagement [36,64]. We found that blocking FcγRIII with specific antibodies could significantly diminish intracellular ROS generation and NET formation. The concentration of anti-FcγRIII antibody (1 or 5 μg/mL) required to suppress the activated dHL-60 is much lower than the concentration (200 μg/mL) for IgG monomer engagement. This result may imply that the binding affinity between a non-specific IgG monomer and FcγRIII is much weaker than that of a specific anti-receptor antibody. Our data strongly suggest that at least partial cross-talk between FcγRIII engagement-induced Syk-ERK signaling pathway and PMA-induced PKC signaling pathway is mediated by ROS that activates PAD4 enzyme activity and NET formation. Another crucial factor in inducing NET formation is the increased intracellular calcium concentration [3,14,16]. Although we did not measure the calcium flux and PAD4 activity in the present investigation, some authors have already reported that the Syk-ERK pathway can increase intracellular ROS generation for activating PAD4 enzyme activity [65,66,67].

In clinical practice, the excessive NET formation contributes to a wide range of disease pathogenesis, including vascular thrombosis, atherosclerosis, autoimmune diseases, and sepsis [19,21,25,26]. As the crucial NET components, DNA, histones, elastase, proteinase 3, myeloperoxidase, and LL-37 are the potential sources of autoantigens participating in the flare-up of autoimmune diseases [8,20,21,22,23]. In recent years, IVIg therapy has become an important option for the treatment of intractable autoimmune diseases [68,69]. Despite the fact that FcγR has been considered an important therapeutic target, only a limited number of investigational drug candidates have been developed for the treatment of autoimmune and inflammatory diseases [70]. We suggest that the elucidation of the effects of IgG engagement on neutrophil biology/pathobiology are crucial for further understanding the molecular basis of autoimmune diseases, inflammatory diseases, and therapeutic rationale of IVIg therapy in future.

## 5. Conclusions

We originally found the engagement of FcγRIII by human monomeric IgG1, IgG2, and the IgG Fc fragment in augmenting the PMA-stimulated NET formation by dHL-60. We further identified that the molecular basis of the augmentation is partially via the cross-talk between the FcγRIII-induced Syk-ERK-NF-κB and the PMA-induced PKC-ROS signaling pathways by increased ROS generation and pro-inflammatory cytokines, IL-8 and TNF-α, production. Whether Syk-ERK signaling can induce intracellular [Ca^2+^] mobilization and PAD4 enzyme activation are now under investigation.

## Data Availability

All figures and data used to support this study are included within this article.
